# Development of the First Quality Assessment Framework for the Endocarditis Multidisciplinary Team Meeting

**DOI:** 10.1093/icvts/ivag219

**Published:** 2026-09-10

**Authors:** Victoria Rizzo, Evgeny Raevsky, Christopher Primus, Ceri Davies, Satya Das, Cristina Suarez, Shirish Ambekar, Neil Roberts, Nandor Marczin, Rakesh Uppal, Kulvinder Lall, Simon Woldman, Thanos Athanasiou, Tayana Soukup

**Affiliations:** Cardiothoracic Surgery, St Bartholomew’s Hospital, Barts-Health NHS Trust, W Smithfield, London, EC1A 7BE, United Kingdom; Department of Surgery and Cancer, Imperial College London, Exhibition Rd, South Kensington, London, SW7 2AZ, United Kingdom; Cardiothoracic Surgery, St Bartholomew’s Hospital, Barts-Health NHS Trust, W Smithfield, London, EC1A 7BE, United Kingdom; Cardiology, St Bartholomew’s Hospital, Barts-Health NHS Trust, W Smithfield, London, EC1A 7BE, United Kingdom; Cardiology, St Bartholomew’s Hospital, Barts-Health NHS Trust, W Smithfield, London, EC1A 7BE, United Kingdom; Microbiology and Infectious Disease, St Bartholomew’s Hospital, Barts-Health NHS Trust, W Smithfield, London, EC1A 7BE, United Kingdom; Microbiology and Infectious Disease, St Bartholomew’s Hospital, Barts-Health NHS Trust, W Smithfield, London, EC1A 7BE, United Kingdom; Cardiothoracic Surgery, St Bartholomew’s Hospital, Barts-Health NHS Trust, W Smithfield, London, EC1A 7BE, United Kingdom; Cardiothoracic Surgery, St Bartholomew’s Hospital, Barts-Health NHS Trust, W Smithfield, London, EC1A 7BE, United Kingdom; Department of Surgery and Cancer, Imperial College London, Exhibition Rd, South Kensington, London, SW7 2AZ, United Kingdom; Cardiothoracic Surgery, St Bartholomew’s Hospital, Barts-Health NHS Trust, W Smithfield, London, EC1A 7BE, United Kingdom; Cardiothoracic Surgery, St Bartholomew’s Hospital, Barts-Health NHS Trust, W Smithfield, London, EC1A 7BE, United Kingdom; Cardiology, St Bartholomew’s Hospital, Barts-Health NHS Trust, W Smithfield, London, EC1A 7BE, United Kingdom; Department of Surgery and Cancer, Imperial College London, Exhibition Rd, South Kensington, London, SW7 2AZ, United Kingdom; Department of Surgery and Cancer, Imperial College London, Exhibition Rd, South Kensington, London, SW7 2AZ, United Kingdom

**Keywords:** endocarditis, multidisciplinary team, quality assessment framework

## Abstract

**Objectives:**

Endocarditis is increasing in incidence with substantial morbidity and mortality. The endocarditis multidisciplinary team (MDT) is a core guideline recommendation with clear patient outcome benefits by expediting diagnosis, coordinating investigations and management. There is significant variability in content and specialist input, with no validated metric available to assess decision-making or demonstrate improvement over time. Multidisciplinary Team Metric for the Observation of Decision-making (MDT-MODe) is a validated quality assessment tool extensively utilized for cancer MDTs. This paper describes the adaptation of the psychometrically tested MDT-MODe to an endocarditis setting, resulting in the development of MDT-MODIE (Metric for the Observation of Decision-making for Infective Endocarditis).

**Methods:**

An expert interview panel was engaged at a single high-volume London centre. Domains were assessed and analysed using a content validity index, through which the final MDT-MODIE was defined. Feasibility testing was conducted during MDTs between September 2024 and April 2025. Inter-rater reliability was assessed using intraclass correlation coefficients (ICCs), while internal validation was evaluated through point-biserial correlation coefficients. Statistical analyses were performed using IBM SPSS Statistics (version 29.0).

**Results:**

Domains scored as excellent or good were included in MDT-MODIE. Feasibility and reliability testing was performed with 152 patient discussions assessed, and scores matched to decision outcomes for each patient. Inter-rater reliability was confirmed across all domains (*P* < .001). Internal validation demonstrated a positive correlation between overall MODIE scores and MDT decision-making outcomes (*r*pb(96) = 0.216, *P *= .033).

**Conclusions:**

Metric for the Observation of Decision-making for Infective Endocarditis provides a reliable framework for evaluating endocarditis MDT decision-making, which has undergone preliminary validation. It addresses a key gap in the literature, supporting cardiac centres in assessing the quality of guideline-recommended MDTs and monitoring improvement over time.

## Graphical Abstract

**Figure:**
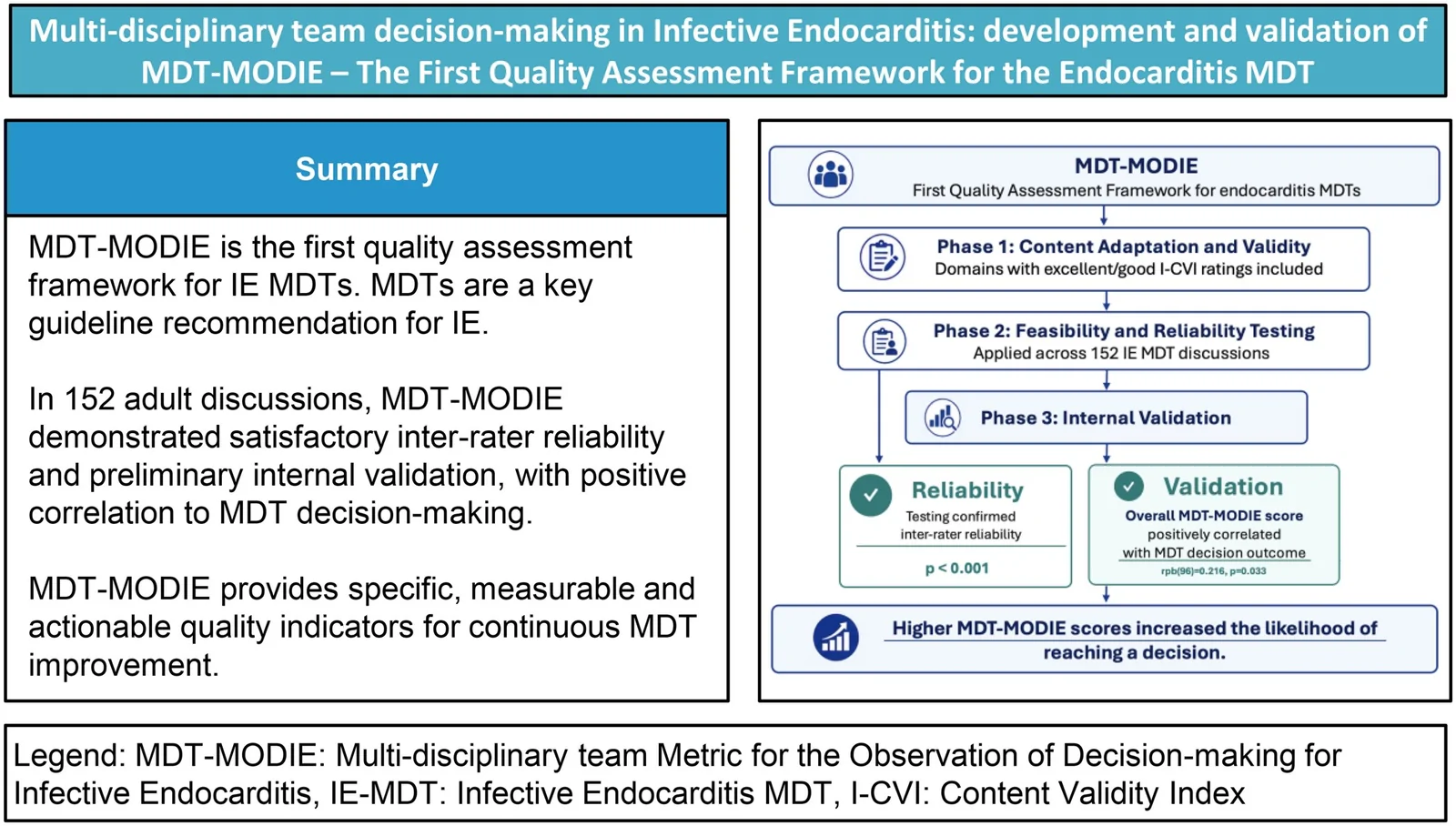

## INTRODUCTION

Infective endocarditis (IE) is characterized by infection of the endocardial surface of the heart, associated with substantial morbidity and mortality. Incidence is increasing, with an estimated at 6-20 cases per 100 000 in high-income countries,^1^ as well as an increasing incidence of prosthetic valve endocarditis (PVE).^2^ Management is complex, requiring prolonged antimicrobial therapy, repeated imaging, and timely consideration of cardiac surgery.

The 2023 ESC (European Society of Cardiology) endocarditis guidelines provide a Class1 recommendation for IE management led by a dedicated multidisciplinary team (MDT), typically comprising cardiology, cardiac surgery, infectious diseases, and imaging specialists.^3^ Where appropriate, other disciplines such as neurology, nephrology, or critical care may be required. In the United Kingdom and Europe, the IE-MDT is increasingly embedded within specialist care pathways, although the structure, composition, and operationalization of these teams vary across settings and are not uniformly mandated or standardized.

The role of the IE-MDT is to expedite diagnosis, coordinate investigations, and decide on an appropriate patient-centred management plan. This includes accurate antimicrobial therapy and assessment of indications for surgery, ensuring optimal timing of intervention.^3^ The IE-MDT is especially important for risk stratification, as there is no widespread effective clinical risk model available for this population.^4^

A clear survival advantage has been demonstrated for patients discussed at an IE-MDT.^5^ Despite this evidence, there is no quality assessment framework for the development and quality control of this MDT. In the absence of a structured quality assessment model, there is limited ability to systematically evaluate MDT functioning, identify variation in decision-making processes, or support consistent improvement over time.

Drawing on the Donabedian model,^6^ the structure and processes of the MDT should consist of specific and measurable process points, with a defined outcome. A successful MDT discussion is defined by the ability to reach a decision, with resultant improved time to adequate treatment or intervention, and improved patient outcomes.

For quality assessment to be meaningful and actionable in practice, tools must be sensitive to the clinical complexity, urgency, and multidisciplinary configuration specific to endocarditis care. The MDT-MODe (Multidisciplinary Team Metric for the Observation of Decision-making) is a quality assessment tool which has been validated and extensively utilized for cancer MDTs.^7^ The aim of this work is to adapt the psychometrically tested MDT-MODe to an endocarditis setting and thus develop a framework for assessing the quality of information and specialist input at the endocarditis MDT.

## METHODS

### Study design and setting

A multi-phase observational instrument development and validation design was used to adapt the existing, psychometrically tested observational assessment tool (MDT-MODe) for use in IE-MDTs. The study comprised 3 sequential phases as described below. The study was conducted at a single high-volume tertiary referral centre within an established IE-MDT in London, United Kingdom. Ethics approval was granted under IRAS-ID249740 (Appendix A).

IE-MDT meeting was held weekly and chaired by Cardiology. Emergency cases were discussed ad hoc with designated specialty leads, ensuring the time-critical decisions were not delayed until the next scheduled MDT.

### Phase1: Content adaptation and expert validation

Adaptation of MDT-MODe for IE was undertaken through structured expert consultation. All specialties in regular IE-MDT attendance were invited to independently rate the relevance of predefined information and specialist input domains to decision-making within the IE-MDT. Ratings were provided using a 4-point relevance scale and assessed using the item-level content validity index (I-CVI) for each domain.

Consistent with established thresholds, I-CVI >0.78 was considered “excellent,” >0.71 “good,” and >0.67 “weak.”^8^ Domains rated as excellent or good were retained in the adapted tool, with weak domains excluded. Experts were also invited to recommend additional criteria. These qualitative suggestions were reviewed by the study team and considered alongside quantitative I-CVI results when finalizing the adapted tool.

The final adapted tool was titled: MDT-MODIE: MDT-Metric for the Observation of Decision-making for Infective Endocarditis.

### Phase 2: Feasibility and inter-rater reliability testing

Feasibility and inter-rater reliability testing were conducted through prospective application of the MDT-MODIE tool to weekly IE-MDTs. Each patient discussion was scored using the tool, with domains rated on a 5-point scale according to the predefined scoring guide. Multidisciplinary team outcome was captured and categorized into 4 mutually exclusive groups:

- Outcome 1: (continued) antimicrobial therapy;
- Outcome 2: surgical intervention;
- Outcome 3: further imaging required; and
- Outcome 4: further information required.

Dichotomy into “decision-reached” (outcomes 1 and 2) and “no-decision-reached” (outcomes 3 and 4) was used for validation analyses, determined by the MDT’s ability to define a management strategy without requiring further input or discussion. The ability to proceed promptly with an agreed treatment plan is essential for timely care with important implications for clinical outcomes, therefore informing our assessment strategy. Outcome 2 was audited for compliance with ESC guidelines.

Thirty percent of patient discussions were independently scored by 2 trained observers (V.R., E.R.) to assess inter-rater reliability. Observers were trained by an expert assessor (T.S.) and blinded to each other’s score. Inter-rater reliability for ordinal domain scores was assessed using intraclass correlation coefficients (ICCs, average measures), with Cronbach alpha reported for internal consistency. An ICC value of >0.7 was indicative of acceptable reliability.^9^ Nominal variables were assessed using Cohen’s kappa, with values >0.81 indicating strong agreement.^10^

In domains with uniform scoring, one-point adjustment of a single data point was made, to enable statistical computation of variance. This was equilibrated by an equal and opposite single-point modification, ensuring consistency with original rater intent and preventing alteration of the substantive interpretation of the ratings.

### Phase 3: Internal validation

Internal validation of the MDT-MODIE tool was assessed by examining the relationship between MDT-MODIE scores and MDT decision outcomes. Point-biserial correlation coefficients were calculated to compare the total MDT-MODIE score (continuous variable) and MDT outcome (decision reached vs no decision reached; dichotomous variable). Correlation strength was interpreted as strong (>0.5), moderate (0.3-<0.5), or small (0.1-<0.3).^11^

### Statistical analysis

Statistical analysis was carried out using the IBM SPSS Statistics Package (v29.0) with significance defined as *P* < .05. Descriptive statistics were used to summarize expert panel characteristics, patient discussions, and MDT-MODIE scores.

## RESULTS

### Phase 1: Content adaptation and expert validation

Eight expert interviews were conducted as follows: cardiologists (*n* = 2), cardiac surgeons (*n* = 2), microbiologists (*n* = 2), a cardiology imaging specialist (*n* = 1), and a specialist nurse (*n* = 1). **Table 1(A)** demonstrates expert panel scoring and I-CVI for *patient-derived information* domains of MDT-MODIE.

**Table 1.** Expert Panel Scoring and I-CVI for the Patient-Derived Information Domains (A) and MDT Input Domains (B) of the MDT-MODIE Tool

MDT-MODIE component	Degree of relevance				CVI	CVI descriptor
	1	2	3	4		
A. *Patient-derived information*						
Patient history				8	1	Excellent
Imaging			1	7	1	Excellent
Microbiology and antibiotics			2	6	1	Excellent
Psychosocial		2	5	1	0.75	Good
Comorbidities			1	7	1	Excellent
Patient views			5	3	1	Excellent
B. *MDT input*						
Cardiology			1	7	1	Excellent
Cardiac surgery				8	1	Excellent
Infectious disease			1	7	1	Excellent
Imaging specialist			5	3	1	Excellent
Specialist nurse		3	3	2	0.625	Very Weak
Pharmacy		3	4	1	0.625	Very Weak

The numbers below the degree of relevance fields represent the number of specialists scoring each variable (*N* = 8 experts).

Abbreviations: CVI, content validity index; MDT, multidisciplinary team; MDT-MODIE, Multidisciplinary Team Metric for the Observation of Decision-making for Infective Endocarditis.

Additional MDT criteria highlighted by experts (n) were as follows: knowledge of patient allergies (2) and need for blood results (2). Risk scoring was also mentioned, although no risk model was specified. While psychosocial information did not receive a unanimous high relevance rating, experts noted that this domain becomes particularly important in certain patient groups, such as intra-venous drug users (IVDUs).

**Table 1(B)** represents expert scoring and I-CVI for the *MDT input domain* of MDT-MODIE. Cardiologist, surgeon, microbiology/infectious disease, and imaging specialist inputs all achieved excellent content validity and were retained in the tool. Nursing and pharmacy inputs were rated as weak and excluded. Additional specialties highlighted by stakeholders included: outpatient parenteral antimicrobial therapy (OPAT) and critical care/anaesthetics (4), although these may only be required for specific cases. During phase 2 testing, an extra field was made available to capture contribution required by an external specialty or specialist nurse.

**Figure 1** depicts the quality assessment process as aligned with the Donabedian model. The final MDT-MODIE tool and guide are depicted in **Figures 2 and 3**.

**Figure 1.**
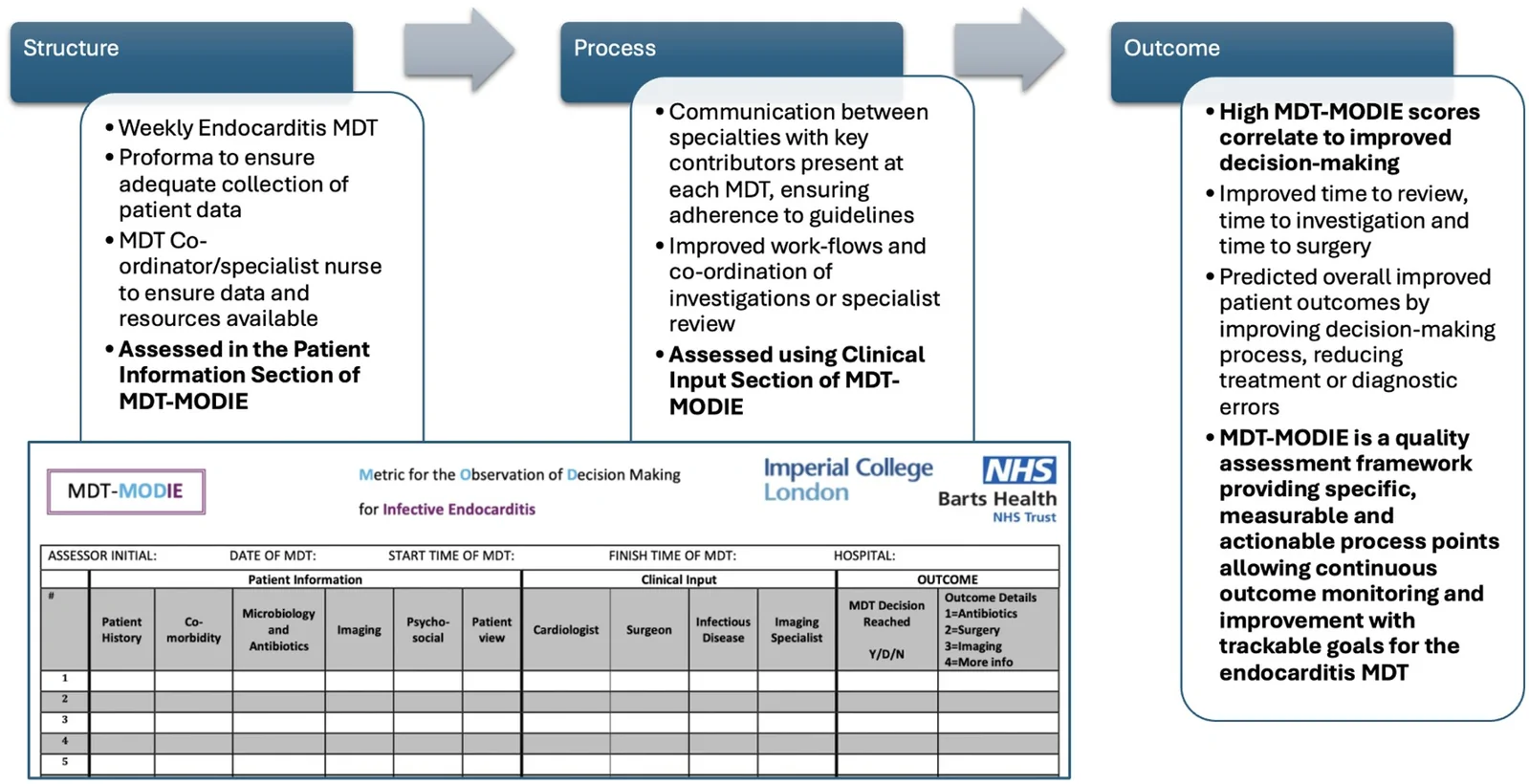
Donabedian Process of Quality Improvement as Related to MDT-MODIE and IE-MDT. Abbreviations: MDT, multidisciplinary team; MDT-MODIE, Multidisciplinary Team Metric for the Observation of Decision-making for Infective Endocarditis

**Figure 2.**
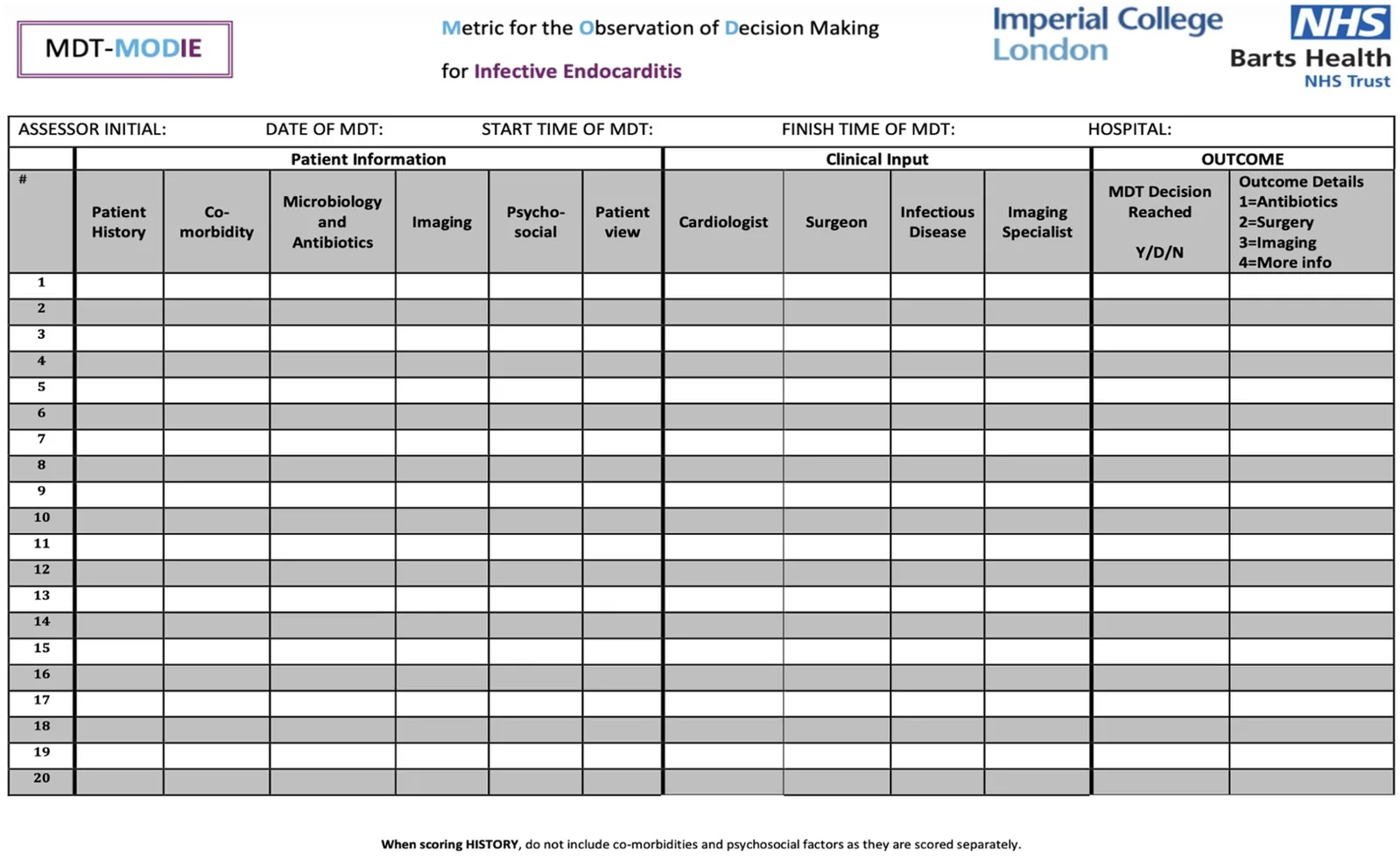
MDT-MODIE Quality Assessment Tool for IE-MDTs. Abbreviations: MDT, multidisciplinary team; MDT-MODIE, Multidisciplinary Team Metric for the Observation of Decision-making for Infective Endocarditis

**Figure 3.**
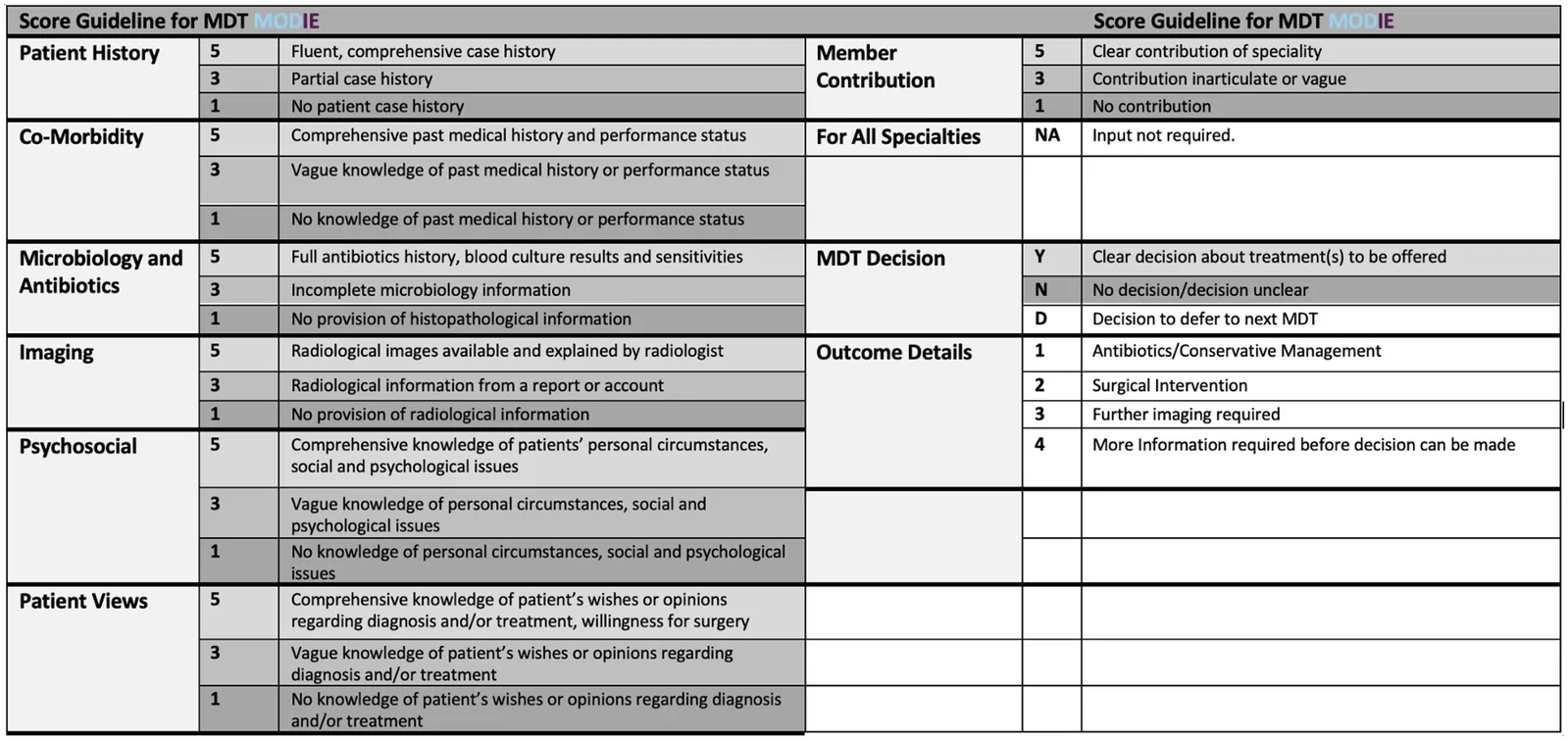
Score Guideline Framework for the MDT-MODIE Tool. Abbreviations: MDT, multidisciplinary team; MDT-MODIE, Multidisciplinary Team Metric for the Observation of Decision-making for Infective Endocarditis

### Phase 2: Feasibility and inter-rater reliability testing

Twelve endocarditis MDT meetings were assessed, with 152 patient discussions included in the analysis. Thirty-two percent (*n* = 37) of cases were independently scored by 2 observers (V.R., E.R.). The mean number of patients discussed in each MDT was 10 (range 6-17). Discussion duration ranged from 29 to 98 minutes per MDT. Time spent per case was analysed for 27 patient discussions, ranging from 1 to 17 minutes, with an average discussion time of 5-8 minutes per patient. Previously discussed cases were generally discussed more briefly.

Patients ranged in age from 19 to 96 years (mean 60.2 ± 1.59). Appendix B summarizes patient demographics and key clinical characteristics. None of the demographic or case complexity variables listed were associated with discussion duration. Organization of the MDT with a detailed proforma (Appendix C) completed by the referring centre and a person with knowledge of the patient being present at the MDT, allowed for efficient discussion with easy representation of critical information in a concise way. This is reflected in the high scores shown across the majority of patient-derived information criteria during this MDT assessment period (Appendix D).

For those requiring surgical intervention, the average time-to-surgery post-MDT was 5 ± 4.2 days (range 1-12). Three further patients were operated semi-electively approximately 27 days later, and 2 were deemed unfit for surgery due to risk. All patients underwent thorough surgical review on the day of the MDT or the following day. The decision to proceed to surgery (outcome 2) was consistent with the 2023 ESC guidelines,^3^ including the recommended timing of surgery.

Specialist nurse input was recorded in 2/115 cases (1.7%). Additional specialist input was required in 8 cases reported here in chronological order: interventional radiology, thoracic surgery, urology, electrophysiology,^2^ dentistry, OPAT, and plastic surgery. Despite the mention of anaesthetic input by 4 key stakeholders in the expert panel review (phase 1), this was not required for any of the patient discussions observed, supporting exclusion as a scored domain. Pharmacy input was not required for any case. Given the low frequency with which these specialties were required, they were not taken forward for further testing or inclusion.

*Inter-rater reliability* is demonstrated in **Tables 2**  **and**  **3**. **Table 2** demonstrates agreement between raters across all domains (*P* < .001). For psychosocial and patient view domains, minimal score variation in quality of input scores was observed, precluding calculation of statistically meaningful ICC estimates. In these domains, quality of input scoring was identical between raters, resulting in ICC and Cronbach alpha values of 1. **Table 3** demonstrates high concordance between observers for nominal MDT-MODIE variables (Cohen’s kappa), including agreement for categorical outcome classifications (*P* < .001).

**Table 2.** Inter-rater Score Comparison (Ordinal Data**)**

Domains		Observer 1		Observer 2		Cronbach alpha	Intraclass correlation coefficient (ICC)		
		Mean	Range	Mean	Range		ICC	95% CI	*P* value
Presentation of patient-derived information	Patient history	4.95	4-5	4.89	3-5	0.855	0.855	0.718-0.925	<.001
	Comorbidity	4.70	3-5	4.78	3-5	0.705	0.705	0.427-0.848	<.001
	Microbiology and antibiotics	4.97	4-5	4.94	4-5	0.795	0.795	0.590-0.898	<.001
	Imaging	4.72	3-5	4.66	3-5	0.925	0.925	0.846-0.963	<.001
	Psychosocial	4.80	4-5	4.80	4-5	1.000	1.000	1.000-1.000	–
	Patient’s view	4.67	4-5	4.67	4-5	1.000	1.000	1.000-1.000	–
Quality of information		20.51		19.30		0.774	0.774	0.561-0.884	<.001
Quality of specialist MDT input	Cardiologist	4.97	4-5	4.97	4-5	1.000	1.000	1.000-1.000	–
	Surgeon	4.95	4-5	4.95	4-5	1.000	1.000	1.000-1.000	–
	Infection	4.90	3-5	4.90	3-5	1.000	1.000	1.000-1.000	–
	Imaging specialist	4.67	4-5	4.67	4-5	1.000	1.000	1.000-1.000	–

Abbreviations: CI, confidence interval; MDT, multidisciplinary team.

**Table 3.** Inter-rater Score Comparison (Nominal Data)

Nominal data	Observer 1		Observer 2		Cohen’s Kappa	Statistical significance
	%Yes	%No	%Yes	%No		*P* value
Patient seen by MDT member	97.3	2.7	97.3	2.7	1.000	<.001
MDT decision reached	89.2	10.8	89.2	10.8	1.000	<.001
Other specialty required	10.8	89.2	10.8	89.2	1.000	<.001
Nurse input required	2.7	97.3	2.7	97.3	1.000	
Outcome reached	% (*n*)		% (*n*)			
Outcome1: Antibiotics	72.97 (27)		72.97 (27)			
Outcome2: Surgery	8.11 (3)		5.41 (2)		0.878	<.001
Outcome3: Imaging	10.81 (4)		10.81 (4)			
Outcome4: More information	8.11 (3)		10.81 (4)		(95% CI 0.714-1.042)	

Abbreviations: CI, confidence interval; MDT, multidisciplinary team.

### Phase 3: Internal validation

Point-biserial correlation analysis demonstrated a positive association between the total MDT-MODIE and MDT decision outcome (*r*pb(96) = 0.216, *P *= .033). This indicates that higher overall MDT-MODIE scores were correlated with an increased likelihood of the MDT reaching a decision. The aggregate score for quality of specialist contribution domains was also positively correlated with decision outcomes (*r*pb (96) = 0.231, *P* = .022). In contrast, the aggregate quality of information score was not significantly associated with MDT decision outcome (*r*pb 96) = 0.139, *P* = .173).

## DISCUSSION

Multidisciplinary teams are the gold standard for clinical decision-making in the United Kingdom,^12^ with their use extending beyond the oncological sphere into other specialities. Literature has repeatedly shown the strength of IE-MDTs in improving patient outcomes, especially by decreasing the rate and lead time to surgical intervention.^5^

A recent meta-analysis, incorporating 15 MDT studies (the first of which was published in 1988), demonstrated significant reduction in short-term mortality after IE-MDT implementation.^5^ A striking feature of this meta-analysis is the variability in MDT team members, the protocol utilized for patient referral, and the regularity of the MDT sessions. Surprisingly, a select number of studies did not have a cardiac surgeon regularly present,^13^^,^^14^ partially reflected in the time-point of referral to the service.

The sole study in this group that failed to demonstrate a significant reduction in short-term mortality was notable for its case-selection protocol. Patients were stratified into complex and non-complex groups, which determined the forum for discussion: conference vs email correspondence.^15^ Despite the lack of statistical significance, after IE-MDT implementation, a reduction in almost 50% of patients requiring unexpected/prolonged ICU admission was noted and surgical intervention increased from 21.6% to 35%.^15^

Although the meta-analysis confirms the efficacy of the IE-MDT, the variability in individual study results and significance, reflects an unmet need for a validated structure and appropriate MDT protocol,^16^ ensuring that all centres can achieve improved survival. This will address an existing gap in the application of evidence-based guidelines purporting the regular use of a structured MDT for this complex group of patients.

Multidisciplinary Team Metric for the Observation of Decision-making has a proven track record in improving the efficacy of oncology MDTs by ensuring the availability of key information and active involvement of essential specialties.^17^^,^^18^ As clinicians face increasing numbers of MDT meetings, MDT-MODe supports accurate and efficient decision-making, reduces the risk of poor performance, and helps prevent decision fatigue by guiding discussions into clear management plans.^18^

Analysis of case complexity factors, for example, the use of MeDiC: Measure of Case-Discussion Complexity tool,^8^ may be a useful next step in IE-MDT assessment. This allows identification of complex cases for priority discussion,^19^ while providing an opportunity for further work-up or external speciality liaison before MDTs. Case prioritization often occurs naturally especially if a patient is critically unwell. Complexity factors have been previously considered, often times excluding more straightforward patients from the traditional MDT discussion.^15^^,^^16^ This is not a validated process and needs further research. Exclusion of certain patients from the formal structure of an MDT may negatively impact individual outcomes.

Although direct contributions were infrequent in this cohort, the specialist nurse played a key organizational role by accruing data, coordinating resources, ensuring attendance of key specialties, and supporting clinical governance within the MDT process. These factors likely underpin the consistently high scores in patient information and clinical domains observed in this study. As such, the MDT-MODIE tool captures measurable decision-making inputs while recognizing that some roles, though essential to MDT functioning, operate upstream of observable discussion dynamics.

This is also applicable to critical liaison services such as OPAT. Decision-making in terms of patient stability and a day 10 trans-oesophageal echocardiogram are the pivotal criteria determining suitability for OPAT.^20^ The MDT remains the ideal forum to accrue all this information in the presence of the necessary specialties for key decision-making and onward liaison with external teams such as OPAT. Psychosocial factors become of increasing relevance in this space, both from a patient compliance point of view, as well as for individuals requiring further social support (eg, IVDU).

Preliminary internal validation demonstrated a modest, but statistically significant, association between higher total MDT-MODIE scores and the likelihood of the MDT reaching a management decision, an association which was primarily driven by the aggregate quality-of-input score. This suggests that while comprehensive information is a necessary foundation for MDT discussion, the effective contribution of key specialist disciplines may be more critical in enabling definitive decision-making. This reinforces the concept that MDT effectiveness depends not only on data completeness, but also on how expertise is mobilized, integrated, and applied during discussion. This correlation provides only preliminary supportive evidence for this model. We recommend application of the model to a larger cohort to further validate and confirm the observed correlation.

The IE-MDT is essential for careful identification of patients requiring early surgery in compliance with guidelines. Timing of surgery is not always straightforward and varies between published guidelines.^3^^,^^21^ Recent literature supports earlier surgery, except in specific cases like intracranial haemorrhage.^22^ The complexity of these cases underscores the importance of specialty-led MDTs, as well as liaison with other specialties. Our results demonstrate short time-to-surgery intervals, with patients undergoing surgery within 24 hours of the MDT. If immediate surgery was not necessary, surgical assessment still occurred within 24 hours.

Another key consideration is the scope of MDT inclusion. Selected studies restricted discussion to selected groups, such as patients with definite rather than possible IE.^23^ Since delayed referral and management are major contributors to poor IE outcomes, we support MDT review of all suspected/possible IE cases, even if some are ultimately not confirmed, as in our cohort. Infective endocarditis diagnosis may be expedited by the MDT, even when not immediately defined using standard criteria,^24^ triggering transfer to a tertiary centre for further assessment at a critical time-point. This broader approach may accelerate time-to-diagnosis and time-to-intervention in definite IE, which are critical to survival. Time-to-advanced imaging such as trans-oesophageal echocardiography, positron emission tomography (PET) and cardiac computed tomography (CT) scanning, may also improve, with the latter being especially useful in patients with PVE.^25^

Limitations of this include single-centre design with need for internal and external validation across multiple centres to confirm wider applicability. Potential observer bias on MDT behaviour must also be considered, despite positive inter-rater reliability analysis. To mitigate potential observer bias, the trained raters were regular attendees, familiar with the subject matter and MDT-members, making it easier to identify information gaps and impaired decision-making. Discussions were also rated independently by the 2 observers, trained by a well-published expert in the field of MDT assessments, to reduce bias.

We note that in certain domains, variance between raters was limited. To mitigate a potential ceiling effect, the development process adopted the use of a 5-point Likert scale (rather than a 3-point scale), along with descriptive rating guides to ensure freedom and accuracy in ratings as recommended by public health experts.^26^^,^^27^ Despite these considerations, we recognize that high ICC values and limited inter-rater domain variance in our study may be a result of the robust and highly structured MDT that has been evaluated. Application to less developed MDTs will be an important step to ascertain discriminatory capacity and internal validation.

Finally, we have utilized a process-based outcome (MDT-decision outcome) as a proxy for MDT effectiveness, and while clinically meaningful, this does not directly capture downstream patient outcomes. Further work linking MDT-MODIE scores to downstream patient outcomes, such as time-to-surgery, length of stay, and mortality, would strengthen understanding of how MDT decision-making quality translates into clinical benefit. Inclusion of compliance analysis with international guidelines would further enhance this work. Published evidence has repeatedly shown that an effective endocarditis MDT translates into improved patient outcomes.

We have shown that MDT-MODIE provides a reliable quality assessment framework for IE-MDTs which has undergone preliminary validation and is easily reproducible. On application of this tool to external IE MDTs, we recommend further internal and external validity assessments, as well as incorporation of downstream patient outcome effects. Ultimately, it is a useful tool for cardiac centres to assess the content and setup of their team meetings and achieve a single decision-making opportunity. Multidisciplinary Team Metric for the Observation of Decision-making for Infective Endocarditis may be particularly useful for less established MDTs, and further testing in less structured settings will be important for further validation and reliability testing. Strengthening of the MDT structure and quality of specialist input will allow for measured and improved MDT outcomes with evidenced ongoing improvement.

## CONCLUSION

We present the first observational assessment tool specifically designed for the IE-MDT, providing a structured framework for adaptation, quality assessment, and continuous improvement of MDT decision-making processes. Evaluation of effective MDT decision-making can be carried out using this standardized MDT-MODIE framework, supported by preliminary validation data. This model will benefit from further multicentre internal and external validation, paired with analysis of downstream clinical outcomes prior to wider application.

The aim of an optimal MDT is to decrease time-to-action, ensure early access to essential interventions, and as a result, improve patient survival. We recommend further application of MDT-MODIE to confirm the internal validation of this tool and allow its use as a quality assessment framework for regulation and standardized benchmarking of this important forum, with the aim of improving patient outcomes through effectual decision-making.

## Supplementary Material

ivag219_Supplementary_Data

## Data Availability

Further data will be shared on reasonable request to the corresponding author.

## RESEARCH APPROVAL

IRAS-ID249740; approved by the NHS Health Research Authority and available at: https://www.hra.nhs.uk/planning-and-improving-research/application-summaries/research-summaries/st-bartholomews-hospital-endocarditis-research-registry/. Further information available in Appendix A.

## ABBREVIATIONS

CVI: content validity index
ESC: European Society of Cardiology
ICC: intraclass correlation coefficient
IE: infective endocarditis
MDT: Multidisciplinary team
MDT-MODe: Multidisciplinary Team Metric for the Observation of Decision-making
MDT-MODIE: Multidisciplinary Team Metric for the Observation of Decision-making for Infective Endocarditis
