# Supplementary material for: Development of the First Quality Assessment Framework for the Endocarditis Multidisciplinary Team Meeting
Source: Interdiscip Cardiovasc Thorac Surg. 2026 Sep 10;41(11):ivag219. doi: 10.1093/icvts/ivag219 (PMC13730559; doi:10.1093/icvts/ivag219)
Supplement: Supplementary file 1 [file ivag219_supplementary_data.docx]

APPENDIX A: Ethics Statement

This study was approved by the Regional Research Ethics Committee IRAS-ID249740, with direct oversight and approval from the Health Research Authority (HRA) and NHS governance processes. The approval encompasses all adult patients referred to the endocarditis MDT at our centre. The requirement for individual patient consent was waived, as discussion within the Endocarditis multidisciplinary team (MDT) formed part of routine clinical care and was necessary for patient management. The primary data collected related to MDT discussion metrics, decision-making processes, and meeting characteristics, rather than direct patient-level interventional data.

Any patient-related information used to contextualise MDT discussions was collected and handled in accordance with the approved protocol, REC/HRA requirements, and relevant UK data governance standards. No additional intervention or change to clinical care was undertaken as part of the study. The collection, storage, and use of study data were conducted in accordance with the approvals granted, including principles consistent with the WMA Declaration of Taipei where applicable.

Registry IRAS- ID 249740 URL: https://www.hra.nhs.uk/planning-and-improving-research/application-summaries/research-summaries/st-bartholomews-hospital-endocarditis-research-registry/

APPENDIX B: Patient Demographics and Pertinent Case Features

| **Characteristics n=110** | **%(n)** |
| --- | --- |
| **Female Gender** | 27.3(30) |
|  |  |
| **Valve Involved:**   - **Aortic** - **Mitral** - **Aortic&Mitral** - **Tricuspid** - **Other (tunnelled venous catheter or unknown)** | 37.3(41)  38.2(42)  10(11)  6.4(7)  8.2(9) |
| **Prosthetic Valve Endocarditis** | 28.2(31) |
| **Cardiac Implantable Electronic Device** | 19.1(21) |
| **Intra-venous drug user** | 2.7(3) |
| **Embolic Phenomenon** | 46.4(51) |
| **Evidence of Intra-Cardiac Destruction** | 16.4(18) |
|  |  |
| **Diabetes Mellitus** | 29.1(32) |
| **Hypertension** | 35.5(39) |
| **Chronic Obstructive Pulmonary Disease** | 8.2(9) |
| **Atrial Fibrillation** | 33.6(37) |
| **LVEF<50%** | 21.8(24) |
| **LVEF<30%** | 4.5(5) |
| Left Ventricular Ejection Fraction: LVEF. | |

Note: *Patients discussed at more than one MDT sitting were only included once unless a new episode of endocarditis identified.*

*Intra-cardiac destruction refers to the structural cardiac damage caused by infective endocarditis, including peri-annular extension, abscess formation, pseudoaneurysm, fistula formation, or prosthetic valve dehiscence.*

APPENDIX C: MDT Proforma

APPENDIX D: MDT-MODIE scoring results across domains during feasibility testing

| Variable | | Mode | Range | |
| --- | --- | --- | --- | --- |
| **Quality of Information Domains** | Patient History | 5 | 3 - 5 | |
|  | Co-morbidity | 5 | 2 - 5 | |
|  | Micro & Abx | 5 | 2 - 5 | |
|  | Imaging | 5 | 1 - 5 | |
|  | Psychosocial | 5 | 1 - 5 | |
|  | Patient View | 5 | 3 - 5 | |
| **Quality of Contribution Domains** | Cardiologist | 5 | 4 – 5 | |
|  | Surgeon | 5 | 2 - 5 | |
|  | Infection | 5 | 1 - 5 | |
|  | Imaging Specialist | 5 | 1 - 5 | |
| Aggregate Scores | | Median | Range | Mean |
| **Aggregate Quality of Information Score** | | 23 (IQR = 20 – 25) | 5 - 30 | 23 ± 5 |
| **Aggregate Quality of Contribution Score** | | 10 (IQR = 10 – 15) | 5 - 20 | 13 ± 4 |
| **TOTAL MDT – MODIE Score** | | 35 (IQR = 30 – 40) | 10 - 50 | 35 ± 8 |

*Note: This table does not report scoring of discussions scored by two independent observers, which are reported elsewhere (Table 2 and Table 3).*

APPENDIX D: Point-biserial Correlation Data (SPSS 29, Pearson)

| Variable 1 | Variable 2 | N | r_pb_ | Correlation Significance | P value |
| --- | --- | --- | --- | --- | --- |
| Quality of Contribution | **OUTCOME (1,2,3,4)** | 98 | .231 | Small | **0.022** |
| Quality of Information | **OUTCOME (1,2,3,4)** | 98 | .139 | None | 0.173 |
| **TOTAL MDT-MODIE Score** | **OUTCOME (1,2,3,4)** | 98 | .216 | Small | **0.033** |
